# Civic League Lectures

**Published:** 1921-02-05

**Authors:** 


### Civic League Lectures.

We have been asked to call attention to several courses, each comprising eight lectures, arranged to be given under the auspices of the Civic Education League this term at Sepley House, Belgrave Road, Westminster. The subjects to be considered are: Civic economics—of special interest to teachers in continuation schools; central and local government; a practising course in the presentment of civics; social psychology; regional survey methods; and the study and teaching of civics.

The lectures started last Monday week.

